# Diagnosis-related differences in the quality of end-of-life care: A comparison between cancer and non-cancer patients

**DOI:** 10.1371/journal.pone.0204458

**Published:** 2018-09-25

**Authors:** Vieri Lastrucci, Sara D’Arienzo, Francesca Collini, Chiara Lorini, Alfredo Zuppiroli, Silvia Forni, Guglielmo Bonaccorsi, Fabrizio Gemmi, Andrea Vannucci

**Affiliations:** 1 Department of Health Science, University of Florence, Florence, Italy; 2 Tuscan Regional Health Agency, Florence, Italy; University of Alabama at Birmingham, UNITED STATES

## Abstract

**Background:**

Cancer, chronic heart failure (CHF), and chronic obstructive pulmonary disease (COPD) in the advanced stages have similar symptom burdens and survival rates. Despite these similarities, the majority of the attention directed to improving the quality of end-of-life (EOL) care has focused on cancer.

**Aim:**

To assess the extent to which the quality of EOL care received by cancer, CHF, and COPD patients in the last month of life is diagnosis-sensitive.

**Methods:**

This is a retrospective observational study based on administrative data. The study population includes all Tuscany region residents aged 18 years or older who died with a clinical history of cancer, CHF, or COPD. Decedents were categorized into two mutually exclusive diagnosis categories: cancer (CA) and cardiopulmonary failure (CPF). Several EOL care quality outcome measures were adopted. Multivariable generalized linear model for each outcome were performed.

**Results:**

The sample included 30,217 decedents. CPF patients were about 1.5 times more likely than cancer patients to die in an acute care hospital (RR 1.59, 95% C.I.: 1.54–1.63). CPF patients were more likely to be hospitalized or admitted to the emergency department (RR 1.09, 95% C.I.: 1.07–1.10; RR 1.15, 95% C.I.: 1.13–1.18, respectively) and less likely to use hospice services (RR 0.08, 95% C.I.: 0.07–0.09) than cancer patients in the last month of life. CPF patients had a four- and two-fold higher risk of intensive care unit admission or of undergoing life-sustaining treatments, respectively, than cancer patients (RR 3.71, 95% C.I.: 3.40–4.04; RR 2.43, 95% C.I.: 2.27–2.60, respectively).

**Conclusion:**

The study has highlighted the presence of significant differences in the quality of EOL care received in the last month of life by COPD and CHF compared with cancer patients. Further studies are needed to better elucidate the extent and the avoidability of these diagnosis-related differences in the quality of EOL care.

## Introduction

The majority of people in high-income countries die of chronic conditions other than cancer [1]. Chronic heart failure (CHF) and chronic obstructive pulmonary disease (COPD) are two prominent causes of death in high-income countries [1, 2], and their prevalence and burden are expected to grow as the populations are aging [2–5]. Although the focus on improving the quality of end-of-life (EOL) care was initially directed mainly to cancer patients [6–9], in recent years awareness of the importance of improving EOL care for patients with non-malignant diseases has increased [10, 11]. Nowadays, there is a general consensus that curative treatment should be integrated with the holistic approach provided by palliative care in the advanced stages of CHF and COPD [12–16].

Cancer, CHF, and COPD at the advanced stages have similar symptom burdens and illness experience [17–19]. They have similar survival rates too [20–22], although prognostication in CHF and COPD is difficult for the less-predictable functional trajectories of these conditions [10, 23]. Despite these similarities, the delivery of quality and comprehensive EOL care for CHF and COPD patients at the advanced stages seems more challenging than for cancer [24]. Indeed, cancer is more likely to be viewed as a terminal condition, whereas CHF and COPD, even in the advanced stages, tend to be treated as chronic conditions [25–28]. Consequently, CHF or COPD patients are less likely to be aware of their prognosis and of the need to provide a ‘Do not resuscitate’ order, and more likely to have unmet palliative care needs [29, 30, 31].

As far as the quality of EOL care is concerned, a few studies have compared patient outcomes across several malignant and non-malignant chronic conditions, with some showing a certain degree of consistency in the patterns of EOL care and treatment intensity provided across conditions [32] and others showing systematic and great diagnosis-related differences [33, 34]. Furthermore, previous studies have focused on treatment intensity outcomes [32] or have been limited to patients of a specific healthcare provider [33, 34].

In order to assess the quality of EOL care, major indicators from administrative data were initially developed and adopted in the context of cancer care [35, 36], and more recently, quality indicators were proposed and adapted to examine the EOL care provided in different serious illnesses and settings [37, 38].

The aim of the present study is to compare the pattern and intensity of EOL care received by cancer, CHF, and COPD patients in the last month of life in order to assess the extent to which the quality of EOL care is diagnosis-sensitive.

## Materials and methods

This is a retrospective observational study of residents in Tuscany based on data extracted from the regional healthcare administrative data system (RHCADS). The RHCADS comprises several healthcare data sources and the following databases were used for the study: enrolment registry, regional census, inpatient hospitalizations, death registry, emergency department (ED), and hospice. In the RHCADS, each resident is represented by a unique encrypted identifier that enables complete record linkage at the level of the individual across databases and over time.

This study was conducted in accordance with the Helsinki Declaration. According to the Italian legislation (law 211/2003) and the regional procedures, the study does not need ethic approval as it is a purely observational study on routine collected anonymous data. Furthermore, because this was an observational retrospective study, patients had already been treated when the study protocol was written; therefore, it could not have modified their life-trajectories or care pathways in any way. Data were extracted from routinely collected administrative databases and there was no need to obtain additional data from individual residents. Data linkage to the patients was performed by using numerical codes, and researchers had access only to an anonymous dataset, which ensured residents’ privacy. For these reasons, no personal informed consent to the present analysis was requested for the study. The Tuscan Regional Health Agency is the authorized public entity (Regional Law n. 40/2005) entitled to perform research and analysis on the RHCADS data.

The study population includes all Tuscany region residents aged 18 years or older who died with a clinical history of cancer, CHF, or COPD in the years 2015 and 2016. More specifically, decedents who had at least one inpatient admission for cancer, CHF, or COPD in the last 36 months of life were included in the study. Because the inclusion criteria was based on inpatient admissions, the last 36-months of life time-frame was chosen in order to minimize the possibility of not including non-hospitalized decedents with cancer, CHF or COPD in the study population. Only decedents who have resided continuously in Tuscany for their last 36 months of life were included in the study as for this population it is possible to ensure complete information for the last 36 months of life. Both primary and secondary diagnosis codes associated with the hospital admissions were used. Table 1 shows in detail the ICD-9-CM inpatient diagnosis codes used.

**Table 1:** Cancer and Cardiopulmonary failure inpatients diagnosis codes (ICD-9-CM) included in the study.

**Cancer**	140–209
**Chronic heart failure**	398.91, 402.01, 402.11, 402.91, 404.01, 404.03, 404.11, 404.13, 404.91, 404.93, 428.XX
**Chronic obstructive pulmonary disease**	491, 492, 493.2, 494, 496, 518.83, 518.84

Decedents who had hospital admissions associated with cancer and cardiopulmonary failure diagnosis codes in the same or in distinct hospitalization episodes were excluded from the study. ED deaths occurring as a consequence of a trauma or injury were excluded from the study; these criteria aimed at excluding people who could not be recognized as being near the end of life until the time of death.

According to the diagnosis codes of the hospitalizations occurred in the last 36 months of life (see Table 1), decedents were categorized into two mutually exclusive diagnosis categories: cancer (CA) and cardiopulmonary failure (CPF). The CPF group includes decedents who had hospital admissions solely associated with diagnoses codes of CHF and/or COPD. Decedents who had hospital admissions solely associated with cancer diagnosis codes were classified in the CA group. CHF and COPD decedents are frequently combined in one category in EOL studies [32, 34] as they have similar patterns of functional decline at the end of life [23].

All the covariates were captured at time of death. The following covariates were considered: age, sex, nationality, education level, comorbidity, and geographic region of residence. Comorbidity was measured using the Charlson comorbidity index (CCI) (Time Frame: last 36 months of life) [39].

The outcome measures adopted in the study were derived from indicators reported to be associated with poor quality of EOL care [35, 37]. Specifically, the outcome measures included: (i) death in an acute care hospital; (ii) one or more hospitalizations in the last month of life; (iii) one or more ED admissions in the last month of life; (iv) one or more intensive care unit (ICU) admissions in the last month of life; (v) use of one or more life-sustaining treatments (LSTs) in the last month of life; and (vi) access to hospice services in the last month of life. The following LSTs were considered: intubation, mechanical ventilation, gastrostomy, haemodialysis, cardiopulmonary resuscitation, pulmonary artery pressure monitoring, implantation of cardiac devices (pacemaker, cardioverter/defibrillator), cardiac catheterization, and surgical intervention.

A descriptive analysis with an χ2 test for categorical and ordinal data and an unpaired t-test for continuous data were performed in order to evaluate significant associations between outcome measures and all the variables considered. A multivariable generalized linear model for binomial distribution for each outcome was performed, including the statistically significant variables resulting from the univariate analyses. Margins for CA and CPF have been computed to estimate adjusted probabilities. For each analysis, an α level of 0.05 is considered as significant. The statistical software Stata 14 SE was used for the data analyses.

## Results

A total of 30,217 decedents with cancer, CHF, and COPD met the eligibility criteria. The patients’ characteristics are presented in Table 2.

**Table 2:** Characteristics of cancer and cardiopulmonary failure cohorts.

	Total	Cancer	CPF	P*
**Total population**	30,217 (100%)	12,159 (40.24%)	18,058 (59.76%)	*< 0*.*001*
**Mean age (Standard Deviation)**	80.47 (11.50)	74.34 (12.37)	84.59 (8.71)	*< 0*.*001*
**Age group**				*< 0*.*001*
*≤ 64*	3,003 (9.93%)	2,421 (19.91%)	582 (3.22%)	
*65–74*	4,381 (14.50%)	2,925 (24.06%)	1,456 (8.06%)	
*75–84*	9,381 (31.05%)	4,103 (33.74%)	5,278 (29.23%)	
*≥ 85*	13,452 (44.52%)	2,710 (22.29%)	10,742 (59.49%)	
**Male**	15,102 (49.98%)	6,425 (52.84%)	8,677 (48.05%)	*< 0*.*001*
**High education level**	5,271 (17.44%)	2,685 (22.08%)	2,586 (14.32%)	*< 0*.*001*
**Foreign nationality**	329 (1.09%)	225 (1.85%)	104 (0.58%)	*< 0*.*001*
**Geographic region**				*< 0*.*001*
*North-western*	10,443 (34.56%)	4,330 (35.61%)	6,113 (33.85%)	
*Central*	13,379 (44.28%)	5,392 (44.35%)	7,987 (44.23%)	
*South-eastern*	6,395 (21.16%)	2,437 (20.04%)	3,958 (21.92%)	
**Charlson Comorbidities Index**				*<0*.*001*
*1–2*	21,706 (71.83%)	9,746 (80.15%)	11,960 (66.23%)	
*3*	5,210 (17.24%)	1,800 (14.80%)	3,410 (18.88%)	
*≥ 4*	3,301 (10.93%)	613 (5.05%)	2,688 (14.89%)	
**Death in acute care hospital**	13,878 (45.93%)	4,432 (36.45%)	9,446 (52.31%)	*< 0*.*001*
**Hospitalizations**	22,619 (74.86%)	8,850 (72.79%)	13,769 (76.25%)	*< 0*.*001*
**ICU admissions**	3,069 (10.15%)	736 (6.05%)	2,333 (12.92%)	*< 0*.*001*
**ED admissions**	18,446 (61.05%)	6,726 (55.32%)	11,720 (64.90%)	*< 0*.*001*
**Use of life-sustaining treatments**	4,331 (14.33%)	1,325 (10.90%)	3,006 (16.65%)	*< 0*.*001*
**Use of hospice services**	2,114 (7.00%)	1,926 (15.84%)	188 (1.04%)	*< 0*.*001*

*Cancer cohort versus CPF cohort

The mean age of the sample is 80.47 years (standard deviation of 11.50 years) and males represent 49.98% of the sample. The CPF cohort represents the 59.76% of the study sample. Upon comparing the characteristics of patients of the two cohorts, patients with CPF were found to be older than the CA patients. The CPF cohort had a higher percentage of patients with a CCI≥3 compared to the CA cohort.

Approximately a third of the CA patients died in acute care hospitals (36.45%), compared with 52.31% of those registered as CPF patients. Among the patients hospitalized in the last month of life, the proportion of patients with one or more ICU admissions or who underwent one or more LSTs was significantly higher in the CPF cohorts (12.92% and 16.65%, respectively) than in the CA cohort (6.05% and 10.9%, respectively). CPF cohort showed a significantly higher proportion of patients with one or more hospital admissions (76.25%) and ED admissions (64.9%) and a lower proportion of patients resorting to hospice services (1.04%) in the last month of life than the CA cohort (72.79%, 55.32%, and 15.84%, respectively).

The variables significantly associated with each outcome of the univariate analyses were included in the multivariable generalized linear model (the univariate analyses for each of the outcome measures are presented in tables A-F in S1 File). Table 3 summarizes the results of multivariable analyses for the two diagnosis cohorts (the results of the full model of multivariable analysis for each outcome measure are reported in tables G-L in S2 File).

**Table 3:** Summary of the results obtained from the multivariable analyses.

	RR	95% C.I.	*P*	Predicted Probabilities	95% C.I.
**Death in acute care hospital**					
*CA cohort (Ref*.*)*	1			34.4	33.5–35.2
CPF cohort	1.59	1.54–1.63	*< 0*.*001*	54.5	53.7–55.3
**Hospitalizations**					
*CA cohort (Ref*.*)*	1			71.2	70.4–72.0
CPF cohort	1.09	1.07–1.10	*< 0*.*001*	77.4	76.8–78.1
**ICU admissions**					
*CA cohort (Ref*.*)*	1			4.5	4.2–4.8
CPF cohort	3.71	3.40–4.04	*< 0*.*001*	16.7	16.1–17.4
**Emergency department admissions**					
*CA cohort (Ref*.*)*	1			56.0	55.0–56.9
CPF cohort	1.15	1.13–1.18	*< 0*.*001*	64.4	63.7–65.1
**Use of life-sustaining treatments**					
*CA cohort (Ref*.*)*	1			8.5	8.0–8.9
CPF cohort	2.43	2.27–2.60	*< 0*.*001*	20.6	19.9–21.3
**Use of hospice services**					
*CA cohort (Ref*.*)*	1			14.5	13.8–15.2
CPF cohort	0.08	0.07–0.09	*< 0*.*001*	1.1	1.0–1.3

Number of observations: 30217

For all the outcomes, the CPF cohort differed significantly from the CA cohort. Specifically, CPF patients were about 1.5 times more likely than CA patients to die in an acute care hospital (RR 1.59, 95% C.I.: 1.54–1.63). Furthermore, in the last month of life, CPF patients were more likely to be hospitalized or admitted to the ED (RR: 1.09, 95% C.I.: 1.07–1.10; RR 1.15, 95% C.I.: 1.13–1.18, respectively) and less likely accessed to hospice services (RR 0.08, 95% C.I.: 0.07–0.09) than CA patients. Among the patients hospitalized in the last month of life, CPF patients have a risk of ICU admission or of undergoing LSTs that is about four- and two-fold higher than for CA patients, respectively (RR 3.71, 95% C.I.: 3.40–4.04; RR 2.43, 95% C.I.: 2.27–2.60, respectively).

## Discussion

The aim of this study was to evaluate whether the quality and treatment intensity of EOL care—measured by several established outcome measures [35–38]—differ significantly among the CA and CPF cohorts. In order to assess the extent to which EOL care is diagnosis-sensitive, several potential determinants and confounders were considered through multivariable analyses. According to the multivariable analyses, the CPF patients differed significantly from the CA patients in all the quality and treatment-intensity EOL care outcomes considered.

Specifically, the study shows that patients with CPF had a higher risk of dying in acute care hospitals and considerably less access to hospice services in the last month of life than patients with CA, which is in line with the literature [33, 34, 40]. Dying in acute care hospitals is a widely recognized indicator of the low quality of EOL care as acute care hospitals are not generally designed to meet the needs of terminally ill patients in terms of symptom control and alleviation [35, 37, 38]. It may be argued that this difference in the place of death may reflect an underlying difference in EOL preferences by diagnosis; however, the literature reports that most CHF and COPD patients prefer to die at home or at non-acute-care care institutions and that these preferences seem to mirror those of patients suffering from malignant diseases [41, 42, 43]. Furthermore, non-malignant patients are reported to experience a higher risk of incongruence between the preferred and actual place of death than cancer patients [44].

In the last month of life, patients with CPF had a higher risk of hospitalization than patients with CA. In the literature, only a few studies have compared the hospitalization rates among cancer and non-cancer patients. In a large retrospective study conducted by Teno et al., a higher hospitalization rate in the last 90 days of life is reported among COPD patients [33]. Conversely, other studies, limited only to the comparison between COPD and lung cancer, have reported a higher hospitalization rate in the last six or 12 months of life among cancer patients [39, 45]. Besides the different patient populations considered in these studies, this apparent discrepancy in the literature may be due to the different time periods in which hospitalization rates are considered and the related potential causes of hospitalization. Indeed, in a more far-from-death period, hospitalization may be more frequently required in cancer patients for potentially appropriate causes (diagnosis establishment and management of chemotherapy and its complications). Conversely, in a period closer to death, these causes of hospitalization occur less frequently and the reasons for hospitalization among patients with different diagnoses potentially becomes more similar [17, 18]. Given the above considerations, the hospitalization rates estimated in a closer-to-death period may serve as a more appropriate indicator for assessing avoidable disparities in the quality of EOL care provided to different diagnosis cohorts.

As far as ED admissions are concerned, the study revealed that CPF groups have a higher risk of ED admission in the last month of life compared to the CA group. A similar result was reported previously [46], although the literature has not devoted much attention to this indicator, and further studies are needed to draw a definite conclusion on this potential disparity.

As for EOL treatment intensity, patients with CPF were at a higher risk of receiving LST or being admitted to the ICU than CA patients. In the literature, differences in EOL treatment intensity between cancer and non-cancer patients have been reported, although there is no general consensus on the extent and magnitude of these differences. A study by Barnato et al. comparing the CA and CHF/COPD cohorts did not find a universally higher EOL treatment intensity in the CHF/COPD cohort [32]. Conversely, other studies reported a systematically lower EOL treatment intensity in CA patients for all the intensity measures analysed [33, 34].

Several factors may explain these differences in EOL care across conditions. First, patients with CHF or COPD may be less frequently involved in patient–physician discussions on EOL matters, in the decision-making process, and in advance care planning. Although the prognosis of COPD and CHF at the advanced stages is poor, physicians may be reluctant to pursue advance care planning with CHF or COPD patients as their illness trajectories are characterized by prognostic uncertainty [23, 47, 48, 49]. Second, advance care planning in the case of CHF and COPD may be hampered by the patient’s and their family’s perceptions of these diagnoses. Indeed, while cancer is often viewed as a terminal condition, COPD and CHF are generally thought of as chronic conditions that are not linked to dying [24, 50]. Lastly, limited clinician knowledge and misperceptions about palliative care as well as a lack of continuity and coordination in healthcare services for CHF or COPD patients may be additional explanations of the registered differences, especially of the lower access to palliative care services in patients with CHF or COPD [51, 52, 53]. For the above reasons, it may be more difficult to recognize impending death and to provide treatments that have aims other than life preservation in COPD or CHF patients.

Nevertheless, several prognostic models, care pathways, clinical guidelines, and recommendations of medical associations have been formulated to assist clinicians to overcome these barriers in patients with end organ failure [12, 14, 24, 47, 54]. In particular, the initiation of palliative care early in the course of the disease in conjunction with curative care is recommended to address patients’ needs holistically, and it may help patients, caregivers, and clinicians to be prepared for the impending death and the transition to less-aggressive treatments near the end of life [55, 56].

Considering that the study population is representative of the deaths occurring in the Tuscany region for CA, CHF, and COPD and that the CPF group represents the majority of the sample, the diagnosis differences in EOL care emerging from this study highlight a relevant issue as well as the need to strengthen EOL care pathways and future research for non-cancer chronic disease and multimorbidity patients. Furthermore, as the prevalence and burden of non-cancer chronic diseases and multimorbidity are expected to grow across many developed countries [2–5], the provision of quality EOL care for non-cancer chronic diseases and multimorbidity patients is an increasingly urgent issue that prompts a call for action.

The sample analysed represents virtually the whole population of an administrative region with around 3.7 million inhabitants and a wide and varied geographical area [57]. Furthermore, the data analysed could be considered as representative of almost all the EOL care that has been provided to the study population as the regional public health system provides healthcare free of charge for the entire population; private providers play a very marginal role in the healthcare system, especially in the provision of EOL care. The above considerations, together with the evaluation of various EOL outcomes measures and confounding factors, should be considered as the strengths of this study.

Nevertheless, the study has several limitations. Firstly, as the study is a retrospective analysis based on administrative data, it may suffer the recognized limitations of using this approach, such as the lack of control on data quality (i.e. underreporting or incorrect reporting of data). However, the quality of administrative data in terms of accuracy and reliability is very high as the data are audited each year. Second, it was not possible to identify and include in the study decedents with cancer or with CHF/COPD who were not hospitalized during the study time-frame; this may have introduced a selection bias. However, it is possible to argue that the extent of this bias was limited as only a very small proportion of decedents with cancer or CHF/COPD was not hospitalized in the their last 36 months of life. Third, a retrospective analysis of administrative data does not allow consideration of important clinical data (e.g. disease severity) or patients’ preferences for care; this could have introduced a bias in the exposure-outcome relationship. Finally, it should be noted that the study relied on inpatients’ diagnosis codes to classify patients in the considered cohorts. This approach is widely adopted in research on EOL matters [23, 33, 34], as several studies have found that the cause of death reported in death certificates is often inaccurate and unreliable [58, 59, 60].

## Conclusions

Cancer, CHF, and COPD in the advanced stages have similar symptom burdens and survival rates. Despite these similarities, the majority of the attention directed to improving the quality of end-of-life (EOL) care has focused on cancer. Although awareness of the quality and aggressiveness of EOL care in CHF and COPD patients has increased recently, research on these issues is still limited. The study has highlighted the presence of significant differences in the quality of EOL care received in the last month of life by COPD and CHF compared with cancer patients. These findings suggest the presence of potential diagnosis-sensitive determinants of the quality of EOL care. Further studies that take into account patients’ preferences of care and disease severity are needed to better elucidate the extent and the avoidability of these differences in the quality of EOL care provided to different diagnosis groups.

## Supporting information

S1 File Univariate analyses for each of the outcome measures. (DOC)

S2 File Full model of multivariable analysis for each outcome measure. (DOC)

S1 Dataset Full dataset of the study. (XLS)

## References

[1] Naghavi M, Abajobir AA, Abbafati C, Abbas K. M., Abd-Allah F, Abera SF, et al Global, regional, and national age-sex specific mortality for 264 causes of death, 1980–2016: a systematic analysis for the Global Burden of Disease Study 2016. The Lancet. 2017; 390(10100):1151–1210. 10.1016/S0140-6736(17)32152-9 PMC5605883 28919116

[2] World Health Organization (WHO). Projections of mortality and causes of death, 2015 and 2030. 2015. http://www.who.int/healthinfo/global_burden_disease/projections/en/ accessed May 22 2018

[3] López-Campos JL, Tan W, Soriano JB. Global burden of COPD. Respirology. 2016; 21:14–23. 10.1111/resp.12660 26494423

[4] Boback Z, Fonarow GC. Epidemiology and aetiology of heart failure. Nature Reviews Cardiology. 2016; 13(6):368–378. 10.1038/nrcardio.2016.25 26935038 PMC4868779

[5] Kontis V, Bennett JE, Mathers CD, Li G, Foreman K, Ezzati M. Future life expectancy in 35 industrialised countries: projections with a Bayesian model ensemble. The Lancet. 2017; 389(10076):1323–1335. 10.1016/S0140-6736(16)32381-9 PMC5387671 28236464

[6] World Health Organization (WHO). Cancer pain relief and palliative care. Report of a WHO Expert Committee. World Health Organ Tech Rep Ser. 1990;804:1–75. 1702248

[7] Higginson I. Palliative care: a review of past changes and future trends. Journal of Public Health. 1993; 15(1):3–8. 10.1093/oxfordjournals.pubmed.a042817 7682424

[8] Stjernswärd J, Colleau SM, and Ventafridda V. The World Health Organization cancer pain and palliative care program past, present, and future. Journal of pain and symptom management. 1996; 12(2):65–72. 8754982 10.1016/0885-3924(96)00109-1

[9] Saunders C. The evolution of palliative care. Patient Education and Counseling. 2000; 41:7–13 10900362 10.1016/s0738-3991(00)00110-5

[10] Curtis JR. Palliative and end-of-life care for patients with severe COPD. European Respiratory Journal. 2008; 32(3):796–803. 10.1183/09031936.00126107 17989116

[11] Goodlin SJ. Palliative care in congestive heart failure. Journal of the American College of Cardiology. 2009; 54(5):386–396. 10.1016/j.jacc.2009.02.078 19628112

[12] Lanken PN, Terry PB, DeLisser HM, Fahy BF, Hansen-Flaschen J, Heffner JE, et al An Official American Thoracic Society Clinical Policy Statement: Palliative Care for Patients with Respiratory Diseases and Critical Illnesses. Am J Respir Crit Care Med. 2008; 177:912–927, 10.1164/rccm.200605-587ST 18390964

[13] Goodridge DM, Marciniuk DD, Brooks D, van Dam A, Hutchinson S, Bailey P, et al End-Of-Life Care for Persons with Advanced Chronic Obstructive Pulmonary Disease: Report of a National Interdisciplinary Consensus Meeting. Canadian Respiratory Journal. 2009; 16(5): e51–e53. 10.1155/2009/987616 19851529 PMC2779173

[14] Yancy CW, Jessup M, Bozkurt B, Butler J, Casey DE, Drazner MH, et al 2013 ACCF/AHA guideline for the management of heart failure: a report of the American College of Cardiology Foundation/American Heart Association Task Force on Practice Guidelines. J Am Coll Cardiol. 2013; 62(16):e147–239. 10.1016/j.jacc.2013.05.019 23747642

[15] Allen LA, Stevenson LW, Grady KL, Goldstein NE, Matlock DD, Arnold RM, et al Decision making in advanced heart failure: a scientific statement from the American Heart Association. Circulation. 2012; 125(15):1928–52. 10.1161/CIR.0b013e31824f2173 22392529 PMC3893703

[16] Fang JC, Ewald GA, Allen LA, Butler J, Westlake Canary CA, Colvin-Adams M, et al Advanced (stage D) heart failure: a statement from the Heart Failure Society of America Guidelines Committee. J Cardiac Fail. 2015;21(6):519–34. 10.1016/j.cardfail.2015.04.013 25953697

[17] Steinhauser KE, Arnold RM, Olsen MK, Lindquist J, Hays J, Wood LL, et al Comparing three life-limiting diseases: does diagnosis matter or is sick, sick?. Journal of pain and symptom management. 2011; 42(3):331–341. 10.1016/j.jpainsymman.2010.11.006 21276704 PMC3597229

[18] Solano JP, Gomes B; Higginson IJ. A comparison of symptom prevalence in far advanced cancer, AIDS, heart disease, chronic obstructive pulmonary disease and renal disease. Journal of pain and symptom management. 2006; 31(1): 58–69. 10.1016/j.jpainsymman.2005.06.007 16442483

[19] Wysham NG., Cox CE, Wolf SP, Kamal AH. Symptom burden of chronic lung disease compared with lung cancer at time of referral for palliative care consultation. Annals of the American Thoracic Society. 2015; 12(9): 1294–1301. 10.1513/AnnalsATS.201503-180OC 26161449

[20] Connors AF Jr, Dawson NV, Thomas C, Harrell FE Jr, Desbiens N, Fulkerson WJ, et al Outcomes following acute exacerbation of severe chronic obstructive lung disease. The SUPPORT Investigators (Study to Understand Prognoses and Preferences for Outcomes and Risks of Treatments) Am J Respir Crit Care Med. 1996;154(4 Pt 1):959–967. 8887592 10.1164/ajrccm.154.4.8887592

[21] McMurray JJ, Stewart S. Epidemiology, aetiology, and prognosis of heart failure. Heart. 2000; 83(5):596–602. 10.1136/heart.83.5.596 10768918 PMC1760825

[22] Stewart S, MacIntyre K, Hole DJ, Capewell S, McMurray JJ. More ‘malignant’than cancer? Five‐year survival following a first admission for heart failure. European journal of heart failure. 2001; 3(3): 315–322. 11378002 10.1016/s1388-9842(00)00141-0

[23] Lunney JR, Lynn J, Foley DJ, Lipson S, Guralnik JM. Patterns of functional decline at the end of life. Jama. 2003; 289(18): 2387–2392. 10.1001/jama.289.18.2387 12746362

[24] Siouta N, van Beek K, Preston N, Hasselaar J, Hughes S, Payne S, et al Towards integration of palliative care in patients with chronic heart failure and chronic obstructive pulmonary disease: a systematic literature review of European guidelines and pathways. BMC palliative care. 2016; 15:18 10.1186/s12904-016-0089-4 26872741 PMC4752742

[25] Lawrence VA, Clark GM. Cancer and resuscitation. Does the diagnosis affect the decision? Arch Intern Med. 1987; 147(9):1637–40. 3632170 10.1001/archinte.147.9.1637

[26] Wachter RM, Luce JM, Hearst N, Lo B. Decisions about resuscitation: inequities among patients with different diseases but similar prognoses. Annals of Internal Medicine. 1989; 111(6): 525–532. 2549825 10.7326/0003-4819-111-6-525

[27] Claessens MT, Lynn J, Zhong Z., Desbiens NA, Phillips RS, Wu AW, et al Dying with lung cancer or chronic obstructive pulmonary disease: insights from SUPPORT. Journal of the American Geriatrics Society. 2000; 48(5 Suppl):S146–53. 10809468 10.1111/j.1532-5415.2000.tb03124.x

[28] Hanson LC, Danis M, Garrett JM, Mutran E. Who decides?: physicians' willingness to use life-sustaining treatment. Archives of Internal Medicine. 1996; 156(7): 785–789. 8615712 10.1001/archinte.156.7.785

[29] Chou WC, Lai YT, Huang YC, Chang CL, Wu WS, Hung YS. Comparing end-of-life care for hospitalized patients with chronic obstructive pulmonary disease and lung cancer in Taiwan. J Palliat Care. 2013; 29(1):29–35. 23614168

[30] Edmonds P, Karlsen S, Khan S, Addington-Hall J. A comparison of the palliative care needs of patients dying from chronic respiratory diseases and lung cancer. Palliative medicine. 2001; 15(4):287–295. 10.1191/026921601678320278 12054146

[31] Gore JM, Brophy CJ, Greenstone MA. How well do we care for patients with end stage chronic obstructive pulmonary disease (COPD)? A comparison of palliative care and quality of life in COPD and lung cancer. Thorax. 2000;55(12):1000–6. 10.1136/thorax.55.12.1000 11083884 PMC1745647

[32] Barnato AE, Cohen ED, Mistovich KA, Chang C-CH. Hospital end-of-life treatment intensity among cancer and non-cancer cohorts. Journal of pain and symptom management. 2015; 49(3):521–529.e1-5. 10.1016/j.jpainsymman.2014.06.017 25135656 PMC4329285

[33] Teno JM, Gozalo PL, Bynum JP, Leland NE, Miller SC, Morden NE, et al Change in end-of-life care for Medicare beneficiaries: site of death, place of care, and health care transitions in 2000, 2005, and 2009. Jama. 2013; 309(5): 470–477. 10.1001/jama.2012.207624 23385273 PMC3674823

[34] Wachterman MW, Pilver C, Smith D, Ersek M, Lipsitz SR, Keating NL. Quality of end-of-life care provided to patients with different serious illnesses. JAMA internal medicine. 2016; 176(8): 1095–1102. 10.1001/jamainternmed.2016.1200 27367547 PMC5470549

[35] Earle CC, Park ER, Lai B, Weeks JC, Ayanian JZ, Block S. Identifying potential indicators of the quality of end-of-life cancer care from administrative data. Journal of Clinical Oncology. 2003; 21(6):1133–1138. 10.1200/JCO.2003.03.059 12637481

[36] Earle CC, Neville BA, Landrum MB, Souza JM, Weeks JC, Block SD, et al Evaluating claims-based indicators of the intensity of end-of-life cancer care. International Journal for Quality in Health Care. 2005; 17(6): 505–509 10.1093/intqhc/mzi061 15985505

[37] Mizuno A, Miyashita M, Hayashi A, Kawai F, Niwa K, Utsunomiya A, et al Potential palliative care quality indicators in heart disease patients: A review of the literature. Journal of Cardiology. 2017; 70(4): 335–341. 10.1016/j.jjcc.2017.02.010 28427868

[38] De Roo ML, Leemans K, Claessen S, Cohen J, Pasman HR, Deliens L, et al Quality Indicators for Palliative Care: Update of a Systematic Review. Journal of pain and symptom management. 2013; 46(4): 556–572. 10.1016/j.jpainsymman.2012.09.013 23809769

[39] Deyo RA, Cherkin DC, Ciol MA. Adapting a clinical comorbidity index for use with ICD-9-CM administrative databases. J Clin Epidemiol. 1992; 45(6):613–9. 1607900 10.1016/0895-4356(92)90133-8

[40] Hyasat K, Sriram KB. Evaluation of the patterns of care provided to patients with COPD compared to patients with lung cancer who died in hospital. American Journal of Hospice and Palliative Medicine. 2016; 33(8): 717–722. 10.1177/1049909115586395 25987648

[41] Beccaro M, Costantini M, Rossi PG, Miccinesi G, Grimaldi M, Bruzzi P, et al Actual and preferred place of death of cancer patients. Results from the Italian survey of the dying of cancer (ISDOC). Journal of Epidemiology & Community Health. 2006; 60(5): 412–416. 16614331 10.1136/jech.2005.043646 PMC2563975

[42] Higginson I, Sen-Gupta. Place of care in advanced cancer: A qualitative systematic literature review of patient preferences. J Palliat Med. 2000; 3(3): 287–300 10.1089/jpm.2000.3.287 15859670

[43] Skorstengaard MH, Neergaard MA, Andreassen P, Brogaard T, Bendstrup E, Løkke A, et al Preferred place of care and death in terminally ill patients with lung and heart disease compared to cancer patients. Journal of palliative medicine. 2017; 20(11): 1217–1224. 10.1089/jpm.2017.0082 28574737

[44] Billingham MJ, Billingham SJ. Congruence between preferred and actual place of death according to the presence of malignant or non-malignant disease: a systematic review and meta-analysis. BMJ Support Palliat Care. 2013; 3(2):144–54. 10.1136/bmjspcare-2012-000292 24644562

[45] Au DH, Udris EM, Fihn SD, McDonell MB, Curtis JR. Differences in health care utilization at the end of life among patients with chronic obstructive pulmonary disease and patients with lung cancer. Archives of Internal Medicine. 2006; 166(3):326–331. 10.1001/archinte.166.3.326 16476873

[46] Smith AK, McCarthy E, Weber E, Cenzer IS, Boscardin J, Fisher J, et al Half of older Americans seen in emergency department in last month of life; most admitted to hospital, and many die there. Health Affairs. 2012; 31(6):1277–1285. 10.1377/hlthaff.2011.0922 22665840 PMC3736978

[47] Coventry PA, Grande GE, Richards DA, Todd CJ. Prediction of appropriate timing of palliative care for older adults with non-malignant life-threatening disease: a systematic review. Age and ageing. 2005; 34(3):218–227. 10.1093/ageing/afi054 15863407

[48] Janssen DJA, Spruit MA, Schols JMGA, Wouters EFM. A call for high-quality advance care planning in outpatients with severe COPD or chronic heart failure. Chest. 2011; 139(5):1081–1088. 10.1378/chest.10-1753 20829337

[49] Curtis JR, Engelberg RA, Nielsen EL, Au DH, Patrick DL. Patient-physician communication about end-of-life care for patients with severe COPD. European Respiratory Journal. 2004; 24(2):200–205. 15332385 10.1183/09031936.04.00010104

[50] Small N, Gardiner C, Barnes S, Gott M, Payne S, Seamark D, et al Using a prediction of death in the next 12 months as a prompt for referral to palliative care acts to the detriment of patients with heart failure and chronic obstructive pulmonary disease. Palliat Med. 2010; 24(7):740–741. 10.1177/0269216310375861 20921093

[51] Kavalieratos D, Mitchell EM, Carey TS, Dev S, Biddle AK, Reeve BB, et al “Not the ‘grim reaper service’”: an assessment of provider knowledge, attitudes, and perceptions regarding palliative care referral barriers in heart failure. Journal of the American Heart Association. 2014; 3(1): e000544 10.1161/JAHA.113.000544 24385453 PMC3959712

[52] Hanratty B, Hibbert D, Mair F, May C, Ward C, Capewell S, et al Doctors' perceptions of palliative care for heart failure: focus group study. Bmj. 2002; 325(7364):581–585. 12228136 10.1136/bmj.325.7364.581 PMC124557

[53] Rodriguez KL, Barnato AE, Arnold RM. Perceptions and utilization of palliative care services in acute care hospitals. Journal of palliative medicine. 2007; 10(1):99–110 10.1089/jpm.2006.0155 17298258 PMC4070316

[54] Nates JL, Nunnally M, Kleinpell R, Blosser S, Goldner J, Birriel B, et al ICU Admission, Discharge, and Triage Guidelines: A Framework to Enhance Clinical Operations, Development of Institutional Policies, and Further Research. Crit Care Med. 2016; 44(8):1553–602. 10.1097/CCM.0000000000001856 27428118

[55] Lynn J, Adamson DM. Living well at the end of life Adapting health care to serious chronic illness in old age. Santa Monica: RAND; 2003.

[56] Murray SA, Kendall M, Boyd K, Sheikh A. Illness trajectories and palliative care. Bmj. 2005; 330(7498):1007–11. 10.1136/bmj.330.7498.1007 15860828 PMC557152

[57] Istituto nazionale di statistica (Istat) [Italian National Institute of Statistics]. Bilancio demografico anno 2016 e popolazione residente al 31 dicembre Regione: Toscana. 2017. http://demo.istat.it/bil2016/index.html. Accessed May 22, 2018

[58] Sehdev AES, Hutchins GM. Problems with proper completion and accuracy of the cause-of-death statement. Archives of Internal Medicine. 2001; 161(2):277–284. 11176744 10.1001/archinte.161.2.277

[59] Pritt BS, Hardin NJ, Richmond JA, Shapiro SL. Death certification errors at an academic institution. Archives of pathology & laboratory medicine. 2005; 129(11):1476–1479. 16253030 10.5858/2005-129-1476-DCEAAA

[60] Ravakhah K. Death certificates are not reliable: revivification of the autopsy. Southern medical journal. 2006; 99(7):728–734. 10.1097/01.smj.0000224337.77074.57 16866055

