# Supplementary material for: Diagnosis-related differences in the quality of end-of-life care: A comparison between cancer and non-cancer patients
Source: PLoS One. 2018 Sep 25;13(9):e0204458. doi: 10.1371/journal.pone.0204458 (PMC6155541; doi:10.1371/journal.pone.0204458)
Supplement: S1 File — (DOC) [file pone.0204458.s001.doc]

**Table A. Univariate analysis of variables associated with death in acute care hospital**

|  | **Death in acute care hospital (%)** | **Total (%)** | ***p*** |
| --- | --- | --- | --- |
| **Sex** |  |  | *< 0.001* |
| Female | 6700 (44) | 15115 (50) |  |
| Male | 7178 (48) | 15102 (50) |  |
| **Age group** |  |  | *< 0.001* |
| *≤ 64* | 1374 (46) | 3003 (10) |  |
| *65-74* | 2059 (47) | 4381 (14) |  |
| *75-84* | 4552 (49) | 9381 (31) |  |
| *≥ 85* | 5893 (44) | 13452 (45) |  |
| **Education level** |  |  | *0.924* |
| *Low* | 11454 (46) | 24946 (83) |  |
| *High* | 2424 (46) | 5271 (17) |  |
| **Nationality** |  |  | *0.920* |
| *Italian* | 13726 (46) | 29888 (99) |  |
| *Foreign* | 152 (46) | 329 (1) |  |
| **Geographic region** |  |  |  |
| *Central* | 6500 (49) | 13379 (44) | *< 0.001* |
| *North-western* | 4741 (45) | 10443 (35) |  |
| *South-eastern* | 2637 (41) | 6395 (21) |  |
| **Charlson Comorbidities Index Score** |  |  | *< 0.001* |
| *1-2* | 9716 (45) | 21706 (72) |  |
| *3* | 2393 (46) | 5210 (17) |  |
| *≥ 4* | 1769 (54) | 3301 (11) |  |
| **Diagnosis** |  |  | *< 0.001* |
| CA cohort | 4432 (36) | 12159 (40) |  |
| CPF cohort | 9446 (52) | 18058 (60) |  |

**Table B.** Univariate analysis of variables associated with hospitalization in the last month of life

|  | **Hospitalization (%)** | **Total (%)** | ***p*** |
| --- | --- | --- | --- |
| **Sex** |  |  | *< 0.001* |
| Female | 11121 (74) | 15115 (50) |  |
| Male | 11498 (76) | 15102 (50) |  |
| **Age group** |  |  | *< 0.001* |
| *≤ 64* | 2388 (80) | 3003 (10) |  |
| *65-74* | 3436 (78) | 4381 (14) |  |
| *75-84* | 7119 (76) | 9381 (31) |  |
| *≥ 85* | 9676 (72) | 13452 (45) |  |
| **Education level** |  |  | *0.615* |
| *Low* | 18659 (75) | 24946 (83) |  |
| *High* | 3960 (75) | 5271 (17) |  |
| **Nationality** |  |  | *0.926* |
| *Italian* | 22372 (75) | 29888 (99) |  |
| *Foreign* | 247 (75) | 329 (1) |  |
| **Geographic region** |  |  | *0.005* |
| *Central* | 10013 (75) | 13379 (44) |  |
| *North-western* | 7907 (76) | 10443 (35) |  |
| *South-eastern* | 4699 (73) | 6395 (21) |  |
| **Charlson Comorbidities Index Score** |  |  | *< 0.001* |
| *1-2* | 16038 (74) | 21706 (72) |  |
| *3* | 3941 (76) | 5210 (17) |  |
| *≥ 4* | 2640 (80) | 3301 (11) |  |
| **Diagnosis** |  |  | *< 0.001* |
| CA cohort | 8850 (73) | 12159 (40) |  |
| CPF cohort | 13769 (76) | 18058 (60) |  |

**Table C.** Univariate analysis of variables associated with ED admission in the last month of life

|  | **Emergency department admission** | **Total (%)** | ***p*** |
| --- | --- | --- | --- |
| **Sex** |  |  | *0.040* |
| Female | 9140 (60) | 15115 (50) |  |
| Male | 9306 (62) | 15102 (50) |  |
| **Age group** |  |  | *< 0.001* |
| *≤ 64* | 1714 (57) | 3003 (10) |  |
| *65-74* | 2510 (57) | 4381 (14) |  |
| *75-84* | 5760 (61) | 9381 (31) |  |
| *≥ 85* | 8462 (63) | 13452 (45) |  |
| **Education level** |  |  | *< 0.001* |
| *Low* | 15365 (62) | 24946 (83) |  |
| *High* | 3081 (58) | 5271 (17) |  |
| **Nationality** |  |  | *0.116* |
| *Italian* | 18259 (61) | 29888 (99) |  |
| *Foreign* | 187 (57) | 329 (1) |  |
| **Geographic region** |  |  | *0.007* |
| *Central* | 8123 (61) | 13379 (44) |  |
| *North-western* | 6493 (62) | 10443 (35) |  |
| *South-eastern* | 3830 (60) | 6395 (21) |  |
| **Charlson Comorbidities Index Score** |  |  | *< 0.001* |
| *1-2* | 12879 (59) | 21706 (72) |  |
| *3* | 3312 (64) | 5210 (17) |  |
| *≥ 4* | 2255 (68) | 3301 (11) |  |
| **Diagnosis** |  |  | *< 0.001* |
| CA cohort | 6726 (55) | 12159 (40) |  |
| CPF cohort | 11720 (65) | 18058 (60) |  |

**Table D.** Univariate analysis of variables associated with ICU admission in the last month of life

|  | **ICU admission (%)** | **Total (%)** | ***p*** |
| --- | --- | --- | --- |
| **Sex** |  |  | *< 0.001* |
| Female | 1302 (9) | 15115 (50) |  |
| Male | 1767 (12) | 15102 (50) |  |
| **Age group** |  |  | *< 0.001* |
| *≤ 64* | 427 (14) | 3003 (10) |  |
| *65-74* | 662 (15) | 4381 (14) |  |
| *75-84* | 1267 (14) | 9381 (31) |  |
| *≥ 85* | 713 (5) | 13452 (45) |  |
| **Education level** |  |  | *0.011* |
| *Low* | 2483 (10) | 24946 (83) |  |
| *High* | 586 (11) | 5271 (17) |  |
| **Nationality** |  |  | *0.227* |
| *Italian* | 3029 (10) | 29888 (99) |  |
| *Foreign* | 40 (12) | 329 (1) |  |
| **Geographic region** |  |  | *< 0.001* |
| *Central* | 1164 (9) | 13379 (44) |  |
| *North-western* | 1258 (12) | 10443 (35) |  |
| *South-eastern* | 647 (10) | 6395 (21) |  |
| **Charlson Comorbidities Index Score** |  |  | *< 0.001* |
| *1-2* | 2161 (10) | 21706 (72) |  |
| *3* | 496 (10) | 5210 (17) |  |
| *≥ 4* | 412 (12) | 3301 (11) |  |
| **Diagnosis** |  |  | *< 0.001* |
| CA cohort | 736 (6) | 12159 (40) |  |
| CPF cohort | 2333 (13) | 18058 (60) |  |

**Table E.** Univariate analysis of variables associated with use of life-sustaining treatments in the last month of life

|  | **Use of life-sustaining treatments (%)** | **Total (%)** | ***p*** |
| --- | --- | --- | --- |
| **Sex** |  |  | *< 0.001* |
| Female | 1910 (13) | 15115 (50) |  |
| Male | 2421 (16) | 15102 (50) |  |
| **Age group** |  |  | *< 0.001* |
| *≤ 64* | 592 (20) | 3003 (10) |  |
| *65-74* | 888 (20) | 4381 (14) |  |
| *75-84* | 1674 (18) | 9381 (31) |  |
| *≥ 85* | 1177 (9) | 13452 (45) |  |
| **Education level** |  |  | *< 0.001* |
| *Low* | 3491 (14) | 24946 (83) |  |
| *High* | 840 (16) | 5271 (17) |  |
| **Nationality** |  |  | *0.086* |
| *Italian* | 4273 (14) | 29888 (99) |  |
| *Foreign* | 58 (18) | 329 (1) |  |
| **Geographic region** |  |  | *< 0.001* |
| *Central* | 1718 (13) | 13379 (44) |  |
| *North-western* | 1743 (17) | 10443 (35) |  |
| *South-eastern* | 870 (14) | 6395 (21) |  |
| **Charlson Comorbidities Index Score** |  |  | *< 0.001* |
| *1-2* | 3121 (14) | 21706 (72) |  |
| *3* | 673 (13) | 5210 (17) |  |
| *≥ 4* | 537 (16) | 3301 (11) |  |
| **Diagnosis** |  |  | *< 0.001* |
| CA cohort | 1325 (11) | 12159 (40) |  |
| CPF cohort | 3006 (17) | 18058 (60) |  |

**Table F.** Univariate analysis of variables associated with the use of hospice services in the last month of life

|  | **Hospice services in the last month (%)** | **Total (%)** | ***p*** |
| --- | --- | --- | --- |
| **Sex** |  |  | *0.431* |
| Female | 1040 (7) | 15115 (50) |  |
| Male | 1074 (7) | 15102 (50) |  |
| **Age group** |  |  | *< 0.001* |
| *≤ 64* | 464 (15) | 3003 (10) |  |
| *65-74* | 557 (13) | 4381 (14) |  |
| *75-84* | 669 (7) | 9381 (31) |  |
| *≥ 85* | 424 (3) | 13452 (45) |  |
| **Education level** |  |  | *< 0.001* |
| *Low* | 1655 (7) | 24946 (83) |  |
| *High* | 459 (9) | 5271 (17) |  |
| **Nationality** |  |  | *0.001* |
| *Italian* | 2075 (7) | 29888 (99) |  |
| *Foreign* | 39 (12) | 329 (1) |  |
| **Geographic region** |  |  | *< 0.001* |
| *Central* | 746 (6) | 13379 (44) |  |
| *North-western* | 1041 (10) | 10443 (35) |  |
| *South-eastern* | 327 (5) | 6395 (21) |  |
| **Charlson Comorbidities Index Score** |  |  | *< 0.001* |
| *1-2* | 1634 (8) | 21706 (72) |  |
| *3* | 357 (7) | 5210 (17) |  |
| *≥ 4* | 123 (4) | 3301 (11) |  |
| **Diagnosis** |  |  | *< 0.001* |
| CA cohort | 1926 (16) | 12159 (40) |  |
| CPF cohort | 188 (1) | 18058 (60) |  |
