# Supplementary material for: Diagnosis-related differences in the quality of end-of-life care: A comparison between cancer and non-cancer patients
Source: PLoS One. 2018 Sep 25;13(9):e0204458. doi: 10.1371/journal.pone.0204458 (PMC6155541; doi:10.1371/journal.pone.0204458)
Supplement: S2 File — (DOC) [file pone.0204458.s002.doc]

**Table G.** Multivariable logistic regression analysis: death in acute care hospital

|  | **Relative**  **Risk** | **95% C.I.** | ***P*** |
| --- | --- | --- | --- |
| **Sex** |  |  |  |
| Female | 1 |  |  |
| Male | 1.04 | 1.01 - 1.06 | *0.002* |
| **Age group** |  |  |  |
| ≤ 64 | 1 |  |  |
| 65-74 | 0.96 | 0.91 - 1.01 | *0.092* |
| 75-84 | 0.90 | 0.86 - 0.94 | *< 0.001* |
| ≥ 85 | 0.74 | 0.71 - 0.77 | *< 0.001* |
| **Geographic region** |  |  |  |
| Central | 1 |  |  |
| North-western | 0.93 | 0.91 - 0.95 | *< 0.001* |
| South-eastern | 0.83 | 0.81 - 0.86 | *< 0.001* |
| **Charlson Comorbidities Index Score** |  |  |  |
| *1-2* | 1 |  |  |
| 3 | 1.00 | 0.97 - 1.03 | *0.975* |
| ≥ 4 | 1.07 | 1.04 - 1.11 | *< 0.001* |
| **Diagnosis** |  |  |  |
| CA cohort | 1 |  |  |
| CPF cohort | 1.59 | 1.54 - 1.63 | *< 0.001* |

Number of observations =30,217

**Table H.** Multivariable logistic regression analysis: Hospitalization in the last month of life

|  | **Relative**  **Risk** | **95% C.I.** | ***P*** |
| --- | --- | --- | --- |
| **Sex** |  |  |  |
| Female | 1 |  |  |
| Male | 1.02 | 1.00 - 1.03 | *0.015* |
| **Age group** |  |  |  |
| ≤ 64 | 1 |  |  |
| 65-74 | 0.97 | 0.95 - 1.00 | *0.022* |
| 75-84 | 0.93 | 0.91 – 0.95 | *< 0.001* |
| ≥ 85 | 0.87 | 0.85 – 0.89 | *< 0.001* |
| **Geographic region** |  |  |  |
| Central | 1 |  |  |
| North-western | 1.00 | 0.99 - 1.02 | *0.510* |
| South-eastern | 0.98 | 0.96 - 0.99 | *0.008* |
| **Charlson Comorbidities Index Score** |  |  |  |
| 1-2 | 1 |  |  |
| 3 | 1.02 | 1.00 - 1.04 | *0.036* |
| ≥ 4 | 1.06 | 1.04 - 1.08 | *< 0.001* |
| **Diagnosis** |  |  |  |
| CA cohort | 1 |  |  |
| CPF cohort | 1.09 | 1.07 - 1.10 | *< 0.001* |

Number of observations =30,217

**Table I**. Multivariable logistic regression analysis: ED admissions in the last month of life

|  | **Relative**  **Risk** | **95% C.I.** | ***P*** |
| --- | --- | --- | --- |
| **Sex** |  |  |  |
| Female | 1 |  |  |
| Male | 1.02 | 1.01 - 1.04 | *0.008* |
| **Age group** |  |  |  |
| ≤ 64 | 1 |  |  |
| 65-74 | 0.96 | 0.92 - 1.00 | *0.077* |
| 75-84 | 1.01 | 0.97 - 1.05 | *0.654* |
| ≥ 85 | 1.01 | 0.97 - 1.04 | *0.781* |
| **Education level** |  |  |  |
| Low | 1 |  |  |
| High | 0.97 | 0.95 - 0.99 | *0.017* |
| **Geographic region** |  |  |  |
| Central | 1 |  |  |
| North-western | 1.02 | 1.00 - 1.04 | *0.039* |
| South-eastern | 0.98 | 0.95 - 1.00 | *0.044* |
| **Charlson Comorbidities Index Score** |  |  |  |
| 1-2 | 1 |  |  |
| 3 | 1.05 | 1.03 - 1.07 | *< 0.001* |
| ≥ 4 | 1.10 | 1.08 - 1.13 | *< 0.001* |
| **Diagnosis** |  |  |  |
| CA cohort | 1 |  |  |
| CPF cohort | 1.15 | 1.13 - 1.18 | *< 0.001* |

Number of observations =30,217

**Table J**. Multivariable logistic regression analysis: ICU admissions in the last month of life

|  | **Relative**  **Risk** | **95% C.I.** | ***P*** |
| --- | --- | --- | --- |
| **Sex** |  |  |  |
| Female | 1 |  |  |
| Male | 1.12 | 1.05 - 1.20 | *0.001* |
| **Age group** |  |  |  |
| ≤ 64 | 1 |  |  |
| 65-74 | 0.88 | 0.79 - 0.98 | *0.017* |
| 75-84 | 0.59 | 0.53 - 0.65 | *< 0.001* |
| ≥ 85 | 0.19 | 0.17 - 0.21 | *< 0.001* |
| **Education level** |  |  |  |
| Low | 1 |  |  |
| High | 1.12 | 1.03 - 1.22 | *0.009* |
| **Geographic region** |  |  |  |
| Central | 1 |  |  |
| North-western | 1.30 | 1.21- 1.40 | *< 0.001* |
| South-eastern | 1.11 | 1.02 - 1.22 | *0.016* |
| **Charlson Comorbidities Index Score** |  |  |  |
| 1-2 | 1 |  |  |
| 3 | 0.92 | 0.84 - 1.01 | *0.071* |
| ≥ 4 | 0.95 | 0.86 - 1.04 | *0.251* |
| **Diagnosis** |  |  |  |
| CA cohort | 1 |  |  |
| CPF cohort | 3.71 | 3.40 - 4.04 | *< 0.001* |

Number of observations =30,217

**Table K.** Multivariable logistic regression analysis: use of life-sustaining treatments in the last month of life

|  | **Relative**  **Risk** | **95% C.I.** | ***P*** |
| --- | --- | --- | --- |
| **Sex** |  |  |  |
| Female | 1 |  |  |
| Male | 1.09 | 1.04 - 1.16 | *0.001* |
| **Age group** |  |  |  |
| ≤ 64 | 1 |  |  |
| 65-74 | 0.91 | 0.83 - 1.00 | *0.042* |
| 75-84 | 0.65 | 0.60 - 0.71 | *< 0.001* |
| ≥ 85 | 0.27 | 0.25 - 0.30 | *< 0.001* |
| **Education level** |  |  |  |
| Low | 1 |  |  |
| High | 1.11 | 1.03 - 1.19 | *0.004* |
| **Geographic region** |  |  |  |
| Central | 1 |  |  |
| North-western | 1.24 | 1.17 - 1.32 | *< 0.001* |
| South-eastern | 1.04 | 0.97 - 1.12 | *0.300* |
| **Charlson Comorbidities Index Score** |  |  |  |
| 1-2 | 1 |  |  |
| 3 | 0.88 | 0.82 – 0.95 | *0.001* |
| ≥ 4 | 0.94 | 0.87 - 1.02 | *0.130* |
| **Diagnosis** |  |  |  |
| CA cohort | 1 |  |  |
| CPF cohort | 2.43 | 2.27 - 2.60 | *< 0.001* |

Number of observations =30,217

**Table L.** Multivariable logistic regression analysis: use of hospice services in the last month of life

|  | **Relative**  **Risk** | **95% C.I.** | ***P*** |
| --- | --- | --- | --- |
| **Age group** |  |  |  |
| ≤ 64 | 1 |  |  |
| 65-74 | 0.93 | 0.83 - 1.05 | *0.234* |
| 75-84 | 0.76 | 0.68 - 0.85 | *< 0.001* |
| ≥ 85 | 0.63 | 0.56- 0.72 | *< 0.001* |
| **Education level** |  |  |  |
| Low | 1 |  |  |
| High | 0.91 | 0.82 - 1.00 | *0.049* |
| **Nationality** |  |  |  |
| Foreign | 1 |  |  |
| Italian | 1.01 | 0.76 - 1.34 | *0.956* |
| **Geographic region** |  |  |  |
| Central | 1 |  |  |
| North-western | 1.74 | 1.59 - 1.89 | *< 0.001* |
| South-eastern | 0.97 | 0.86- 1.10 | *0.611* |
| **Charlson Comorbidities Index Score** |  |  |  |
| 1-2 | 1 |  |  |
| 3 | 1.12 | 1.01 - 1.24 | *0.036* |
| ≥ 4 | 0.96 | 0.81 - 1.15 | *0.683* |
| **Diagnosis** |  |  |  |
| CA cohort | 1 |  |  |
| CPF cohort | 0.08 | 0.07 – 0.09 | *< 0.001* |

Number of observations =30,217
